# Vanin 1: Its Physiological Function and Role in Diseases

**DOI:** 10.3390/ijms20163891

**Published:** 2019-08-09

**Authors:** Roberta Bartucci, Anna Salvati, Peter Olinga, Ykelien L. Boersma

**Affiliations:** 1 Division of Pharmacokinetics, Toxicology and Targeting, Groningen Research Institute of Pharmacy, University of Groningen, A. Deusinglaan 1, 9713 AV Groningen, The Netherlands; 2 Division of Pharmaceutical Technology and Biopharmacy, Groningen Research Institute of Pharmacy, University of Groningen, A. Deusinglaan 1, 9713 AV Groningen, The Netherlands; 3 Division of Chemical and Pharmaceutical Biology, Groningen Research Institute of Pharmacy, University of Groningen, A. Deusinglaan 1, 9713 AV Groningen, The Netherlands

**Keywords:** vanin 1, oxidative stress, PPAR-alpha, PPAR-gamma, urinary biomarker, pro-inflammatory role, protective role

## Abstract

The enzyme vascular non-inflammatory molecule-1 (vanin 1) is highly expressed at gene and protein level in many organs, such as the liver, intestine, and kidney. Its major function is related to its pantetheinase activity; vanin 1 breaks down pantetheine in cysteamine and pantothenic acid, a precursor of coenzyme A. Indeed, its physiological role seems strictly related to coenzyme A metabolism, lipid metabolism, and energy production. In recent years, many studies have elucidated the role of vanin 1 under physiological conditions in relation to oxidative stress and inflammation. Vanin’s enzymatic activity was found to be of key importance in certain diseases, either for its protective effect or as a sensitizer, depending on the diseased organ. In this review, we discuss the role of vanin 1 in the liver, kidney, intestine, and lung under physiological as well as pathophysiological conditions. Thus, we provide a more complete understanding and overview of its complex function and contribution to some specific pathologies.

Vascular non-inflammatory molecule-1, in short vanin 1, is an ectoenzyme with pantetheinase activity [1]. It is highly expressed at the gene and protein level in many organs, such as the liver, intestine, and kidney. Its major function is related to its pantetheinase activity, as follows: Vanin 1 breaks down pantetheine into cysteamine and pantothenic acid, a precursor of coenzyme A. Indeed, its physiological role seems strictly related to coenzyme A metabolism, lipid metabolism, and energy production. In recent years, many studies have elucidated the role of vanin 1 under physiological conditions in relation to oxidative stress and inflammation. Vanin’s enzymatic activity was found to be of key importance in certain diseases, either for its protective effect or as a sensitizer, depending on the diseased organ. In this review, we discuss the role of vanin 1 in the liver, kidney, intestine, and lung under physiological as well as pathophysiological conditions. Thus, we provide a more complete understanding and overview of its complex function and contribution to some specific pathologies.

Vanin 1 was first identified as a novel surface molecule involved in thymus homing of bone marrow cells in mice; yet, sequence analysis of vanin 1 showed no homology with other adhesion proteins [1]. Two human counterparts of the murine Vnn1 gene (m*Vnn1*), h*Vnn1*, and h*Vnn2*, were identified later, sharing 78% and 64% of sequence identity with m*Vnn1*, respectively [2]. Recently, the 3D structure of human vanin 1 confirmed the presence of a nitrilase domain conferring enzymatic activity (PDB code 4CYF) [3]. In addition, the structure revealed the presence of a base domain which could be involved in cellular adherence and homing through protein–protein interactions. Though we do not exclude a potential role for the base domain as a signaling protein, here we focus on vanin’s pantetheinase activity in different organs.

Vanin 1 is membrane-associated via a glycosylphosphatidylinositol (GPI)-anchor, though soluble forms of human and mouse vanin 1 have been detected as well [4]. Under physiological conditions, the mRNA expression of human vanin 1 is particularly high in lung, liver, kidney, gastrointestinal tract (mainly in the duodenum and jejunum [5]), spleen, blood, and skin [6]. In murine tissues, the gene expression of vanin 1 is found to be high mainly in the kidney, intestine, and liver [7,8]. hVanin 2 is a GPI-anchored pantetheinase as well, expressed in tissues such as spleen, kidney, and lungs; however, hVanin 2 shows a much higher expression in the blood than hVanin 1 [6], specifically in neutrophils [8,9,10]. Scanning general and organism-specific expressed sequence tag (EST) databases led to the identification of *hVnn3*, which maps to the human chromosome 6q22-24 region, as do *hVnn1* and *hVnn2* [9,11]. *hVnn3* appears to encode a truncated protein, suggesting it might be a pseudogene or encoding a protein other than a pantetheinase [8]. Other vanin molecules include mVanin 3, a rat vanin 1 orthologue, a drosophila homologue, and bottlenose dolphin vanin 1 [11,12]. mVanin 3 mRNA has been detected in lung tissue, liver, and blood, while its gene expression is completely absent in kidney [6,8,9]. Among these genes belonging to the pantetheinase family, murine and human vanin 1 have been studied best. Here, we provide an overview of vanin 1′s role in physiological and pathophysiological conditions in different organs.

## 1. Function, Expression, and Regulation of Vanin 1 Under Physiological Conditions

Vanin 1′s primary function is the recycling of pantothenic acid (vitamin B5), an important precursor in the biosynthesis of CoenzymeA (CoA); indeed, vanin 1 is highly expressed in tissues with a high CoA turnover, such as liver, intestine, and kidney [13]. CoA, the cofactor known for its role in the synthesis and oxidation of fatty acids [14], is assembled in five steps from pantothenic acid, cysteine and adenosine triphosphate (ATP) [15]. CoA homeostasis is regulated by its biosynthesis, degradation, and the use of free CoA as a conjugate, e.g., as acyl-CoA. While CoA biosynthesis has been studied in detail, relatively little is known about CoA degradation, though a number of enzymes (e.g., nudix hydrolases) are hypothesized to play a role [14,15]. These enzymes consequently regulate the bioavailability of phosphopantetheine, which in turn is degraded through phosphatase and pantetheinase activity [16]. This specific enzymatic activity was first observed in crude horse kidney extracts, in which the oxidized form of pantetheine, pantethine, was converted into pantothenic acid and cysteamine [17,18]. Pantetheinase activity was also observed in other organs of other mammals, such as birds, pigs, and rats. Based on sequence homology with pig kidney pantetheinase, vanin 1 was identified as a pantetheinase [19].

**Figure 1:**
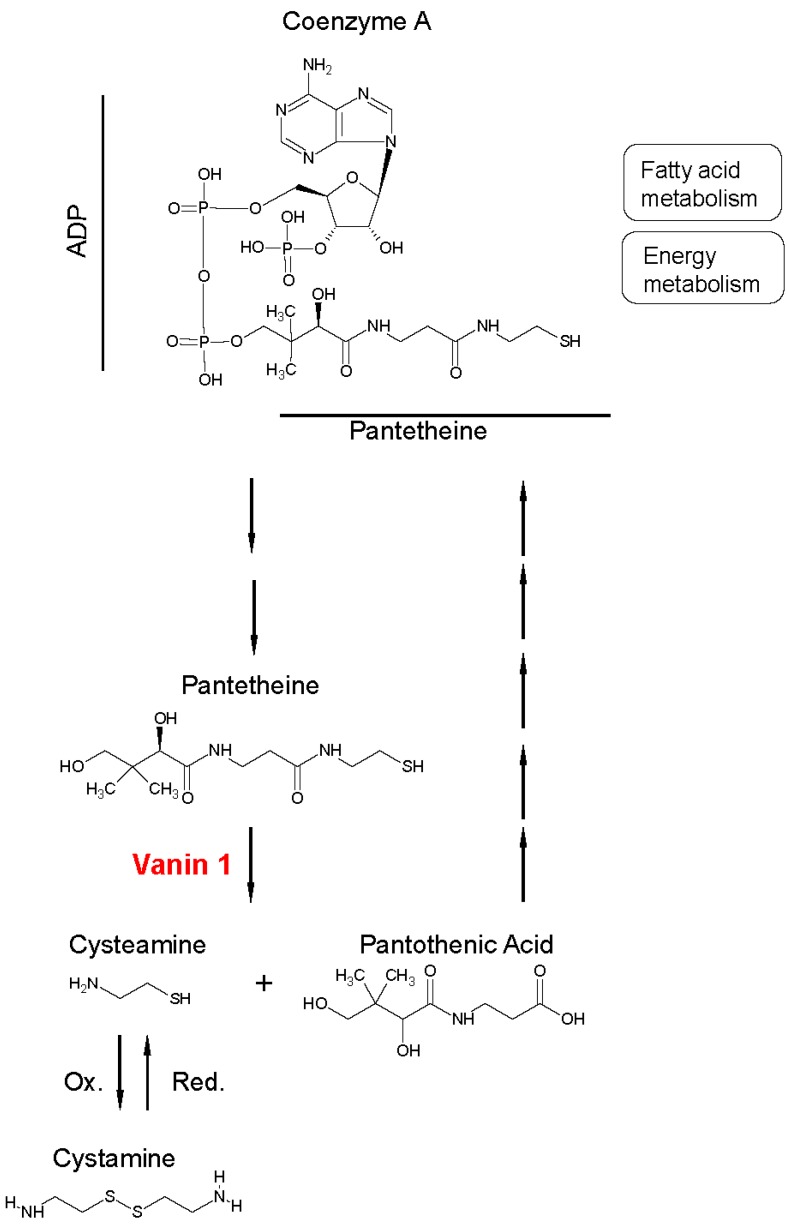
Schematic overview of vanin 1′s enzymatic activity. Vanin 1′s substrate is pantetheine, which makes up the structure of coenzyme A (CoA), together with adenosine diphosphate (ADP). The enzyme catalyzes the hydrolysis of pantetheine into pantothenic acid and cysteamine, which in turn can be oxidized (Ox.) to cystamine. Pantothenic acid can be recycled as a precursor of CoA after five biochemical reaction steps (symbolically represented by five arrows).

During CoA catabolism, as illustrated in Figure 1, vanin 1 specifically hydrolyzes one of the amide bonds of the substrate pantetheine into two products, pantothenic acid and cysteamine, which are considered potent antioxidants [20]. The formation of pantothenic acid allows for a continuous production of CoA, as pantothenic acid is a structural component of this cofactor. In addition, pantothenic acid appears to have profibrotic effects, being involved in the promotion of proliferation and migration of dermal fibroblasts [21,22,23]. Moreover, pantothenic acid contributes to restoring CoA levels in the mitochondria, resulting in enhanced mitochondrial activity [24].

Cysteamine, an aminothiol, can be oxidized to the disulfide cystamine. This reaction is reversible (Figure 1). Together, cysteamine and cystamine are important sensors of oxidative stress, maintaining the thiol-disulfide equilibrium through protein disulfide exchange [25,26]. At low concentrations, cysteamine can form a complex with cysteine, promoting the latter’s transport into cells. Cysteine is subsequently incorporated into the strong antioxidant glutathione (GSH) by the enzyme γ-glutamylcysteine synthetase (γ-GCS). High concentrations of cysteamine inhibit the activity of glutathione peroxidase, which catalyzes the oxidation of GSH to glutathione disulfide (GSSG) to maintain the intracellular redox homeostasis [27,28]. In addition, γ-GCS is inhibited by high concentrations of cystamine, thus depleting the intracellular stores of GSH [29]. The role of cysteamine and cystamine is illustrated by experiments using *vnn1*–deficient mice. The absence or reduced level of cysteamine led to an enhanced γ-GCS activity and, consequently, led to elevated endogenous GSH stores in tissues [7]. This conferred a higher resistance to oxidative stress exposure to *vnn1* knockout (KO) mice compared to wild type (WT) mice; the resistance could be abolished by administration of cystamine, likely due to inhibition of γ-GCS through protein disulfide exchange [30,31]. As a consequence, *vnn1*-KO mice were better protected against tissue inflammation in response to systemic oxidative stress, suggesting that vanin 1 may promote inflammation. 

It has been shown that vanin 1 also plays a role in the regulation of a number of metabolic pathways. In fact, the *vnn1* gene was found to be one of the positive targets of the peroxisome proliferator-activated receptor alpha (PPAR-α) in mouse liver [4,32,33]. PPAR-α is a key regulator of the liver’s response to fasting. It promotes uptake, utilization, and catabolism of fatty acids by upregulation of genes involved in fatty acid transport, fatty acid activation, and peroxisomal and mitochondrial fatty acid β-oxidation [34]. Microarray analyses showed that, upon administration of the PPAR-α agonists fenofibrate, clofibrate, or Wy-14,643, *vnn1* expression increased [35,36]. In line with these findings, PPAR-α KO mice showed a negligible liver *vnn1* expression [37,38], even after stimulation with various agonists. [33]. Rommelaere et al. used a bioinformatics approach to confirm PPAR-α’s regulation of *vnn1* gene expression. Two candidate sites for PPAR-α response elements (PPRE) were identified upstream of *vnn1.* Focusing on the proximal site, reporter assays using constructs containing parts of the vanin 1 promoter upstream of the luciferase gene were carried out. Following transfection, luciferase activity was significantly induced in the presence of PPAR-α agonists when using a 3.5 kb part of the vanin 1 promoter. Sequence analysis identified two PPRE sites within the vanin 1 promoter region. In addition, serum vanin 1 levels in the liver were shown to be regulated by PPAR-α activity. Treatment with the PPAR-α agonist fenofibrate resulted in an increased production of serum vanin 1 [4]. These levels of serum vanin 1 might be due to altered expression ascribed to polymorphism in regulatory regions [39].

*vnn1* has been found to directly regulate PPAR-γ mRNA expression in gut epithelial cells [40]. PPAR-γ regulates energy storage [41] and plays an important role in the innate and adaptive immune response. This receptor has long been studied for its anti-inflammatory activity. Berruyer et al. showed that *vnn1* prevents PPAR-γ nuclear translocation; as such, the presence of *vnn1* is key for the perception of stress by innate immune cells. 

PPAR-γ also controls the expression of many genes related to the regulation of adipocyte differentiation, fatty acid (FA) storage, and glucose metabolism. In a study by Chen et al. [42], vanin 1 was shown to be an activator of hepatic gluconeogenesis in mice. Vanin 1 overexpression increased glucose output by specifically increasing the hepatic transcription levels of gluconeogenic genes, while *vnn1* knockdown decreased the glucose output of murine hepatocytes. The transcription of *vnn1* itself was activated by PPAR-γ coactivator 1α (PGC-1α) in complex with the hepatocyte nuclear factor-4α (HNF-4α). In turn, this complex is mediated by the Akt signaling pathway. In fact, the insulin-Akt signaling axis plays an important role in regulating the gluconeogenesis and is activated by PPAR-γ [26,27]. Upon PGC-1α overexpression, both in vitro and in vivo, an increased level of vanin 1 expression at both the gene and the protein level was observed, suggesting *vnn1* is a direct target for PGC-1α. Indeed, when PGC-1α gene expression was knocked down, a decrease of vanin 1 expression in the liver and in cultured hepatocytes was observed. Thus, PGC-1α positively regulates *vnn1* gene expression, which in turn regulates gluconeogenesis. In contrast, vanin 1′s reaction products cysteamine and pantothenic acid are involved in regulating glycolysis. Cysteamine was reported to play a role in limiting glycolysis and lactate release [24], while pantothenic acid was linked to high lactate release, indicating glycolytic activity [43].

## 2. Vanin 1 Expression and Its Role in Diseases

In recent years, a number of studies have identified vanin 1 as a key player in the development and continuation of diseases, in both animal models and humans. These roles of vanin 1 will be discussed below with regard to the different organs in which the enzyme is expressed.

### 2.1. Liver

In both mouse and human liver, vanin 1 is expressed at the gene and protein levels [7,37,42,44,45]. The protein vanin 1 is expressed by hepatocytes, the major cell type of the liver, specifically by the centrilobular hepatocytes in zone 3 adjacent to the central vein. These hepatocytes are involved in lipid and xenobiotic metabolism [46]. Both processes are regulated by PPAR-α activation [47].

#### 2.1.1. Vanin 1 in Steatosis, NAFLD, and NASH Models

Hepatic vanin 1 expression and activity were previously shown to be significantly induced by dietary fatty acids [38]. The *vnn1* gene being a target of PPAR-α [4], it comes as no surprise that oral administration of PPAR-α agonists such as fenofibrate significantly induced vanin 1 expression [32,33]. PPAR-α contributes to liver homeostasis by limiting steatosis; however, the association of vanin 1 with hepatic steatosis is somewhat ambiguous. Some mouse studies showed a strongly increased vanin 1 expression in high-fat diet-induced steatotic livers [37,48]. This would imply a causal role for vanin 1 in the progression of steatosis. On the other hand, Van Diepen et al. [33] observed that the presence of vanin 1 actually protects against the development of steatosis induced by prolonged starvation in mice. Using *vnn1* KO and WT mice, they showed that vanin 1 deficiency led to an increased accumulation of lipids and hepatic triglyceride (TG) levels in 24 h fasted livers. In addition, when rats were treated with the vanin 1 inhibitor RR6 [49] for four days and subsequently fasted for 24 h, they showed an increased liver weight, indicating increased hepatic steatosis. Furthermore, vanin 1 activity in human plasma was analyzed. The activity was found to be increased by PPAR-α activation, induced by fasting or fibrate treatment. These findings suggest that the relationship between steatosis and vanin 1 expression may be explained by an increased activation of PPAR-α upon fasting and subsequent transcription of its target genes in steatotic livers.

Non-alcoholic fatty liver disease (NAFLD) is characterized by an increased fat deposit in the liver and the accumulation of saturated free fatty acids (FFAs), which contribute to disease progression through toxic effects on hepatocytes [50]. Indeed, lipotoxicity may result in hepatocyte damage, triggering an inflammatory reaction and an abnormal wound healing response that results in the development of non-alcoholic steatohepatitis (NASH) and fibrosis [51,52]. Growing evidence suggests that angiogenesis plays a central role in the progression to NASH [53,54,55,56]. During liver fibrogenesis, hepatic stellate cells (HSCs) switch from being quiescent to activated and proliferative myofibroblast-like cells. This activation involves up-regulation of various genes, including α-smooth muscle actin (α-SMA), collagen-1α1, and transforming growth factor-β (TGF-β) [57]. Povero et al. mimicked lipid accumulation that occurs in NAFLD and NASH and showed that hepatocytes secreted microparticles (MPs), both in vitro and in vivo [44,45]. Upon internalization, these MPs promoted angiogenesis. LC-MS/MS analysis showed that vanin 1 was one of the most abundant surface proteins in hepatocyte-derived MPs. Next, MPs were exposed to human umbilical vein endothelial cells (HUVECs). Since GPI-anchored proteins are often associated with lipid raft domains in the plasma membrane, immunogold electron microscopy studies were undertaken to examine the colocalization of vanin 1-positive MPs and HUVEC lipid rafts to assess whether the internalization of microparticles could be promoted by vanin 1. In fact, vanin 1-positive MPs were found to be colocalized with the HUVEC lipid rafts. To independently confirm these results, a vanin 1 neutralizing antibody was used to block the interaction of the lipid rafts and vanin 1, and a significant reduction in MP uptake, cell migration, and tube formation was observed. Furthermore, MPs derived from hepatocytes were found to carry and transfer microRNAs (miRNA, e.g., miR-128-3p) that regulate fibrogenesis by inducing a phenotypical switch from a quiescent state to activated HSCs [45]. Proteomics analysis showed that the MPs were enriched in vanin 1 on the external leaflet and that vanin 1 was, again, required for the interaction with lipid raft domains. Thus, vanin 1 forms an important link between lipid accumulation and hepatic diseases, such as fibrosis.

#### 2.1.2. Vanin 1 and Hepatotoxicity

Vanin 1 has also been studied in relation to hepatotoxicity, for instance drug-induced toxicity. APAP (acetyl-*para*-aminophenol, or paracetamol) is one of the most commonly used drugs, owing mainly to its safety profile. However, when taken in large amounts, APAP can lead to severe necrosis in the centrilobular hepatocytes [43,58]. It was previously demonstrated in mouse models that vanin 1 protects the liver from hepatotoxicity, specifically from APAP injury [59,60,61]. Mice lacking vanin 1 were shown to be sensitive to APAP hepatotoxicity and they exhibited increased concentrations of plasma alanine aminotransferase (ALT) and more necrosis compared to WT mice. Importantly, the susceptibility of vanin 1 KO mice to APAP hepatotoxicity was not due to a decreased capacity of the liver to detoxify APAP via GSH, though lowered proliferative and immune responses were observed [60]. As a consequence, compared to WT mice, less infiltration of immune cells within areas of centrilobular necrosis was seen in *vnn1* KO mice, leading to even more severe damage. Furthermore, vanin 1 KO mice showed higher susceptibility to hepatic injury, also when other hepatotoxicants such as carbon tetrachloride (CCl_4_) and concanavalin A were administered. Thus, the damage reported was not unique to APAP. Moreover, the observation of a lowered immune response in *vnn1* KO mice could suggest a correlation between immune cells and the expression of vanin 1 [60]. Within this context, vanin 1 seems to play a protective role when the liver is considerably injured.

Kisseleva and coworkers evaluated the origin of hepatic myofibroblasts in WT C57BL/6 mice after chronic injury with either CCl_4_ or common bile duct ligation (BDL) [62]. Hepatotoxic (CCl_4_) and cholestatic (BDL) injuries were shown to activate distinct subsets of fibrogenic myofibroblasts. Interestingly, *vnn1* expression also varied depending on the etiology of the hepatic injury. With the use of a whole mouse genome microarray, *vnn1* expression was found to be increased 8.5-fold in activated portal fibroblasts (aPFs) obtained via a BDL fibrotic mouse model, compared to activated hepatic stellate cells (aHSCs) obtained by BDL and CCl_4_ models [62]. These findings suggest that *vnn1* expression and regulation can strictly depend on the specific diseased liver model and, in this particular case, also on the origin of myofibroblasts.

### 2.2. Kidney

In physiological conditions, mouse, rat and human vanin 1 are expressed in the brush borders of the proximal tubuli of the nephron [7]. These proximal tubular cells are characterized by the presence of microvilli and, therefore, they present an increased apical surface area in which many transporters and channels are expressed [63]. The presence of vanin 1 specifically at the brush borders suggests that vanin 1 is pivotal for the salvaging and recycling of pantothenic acid.

#### 2.2.1. Vanin 1 in Acute Kidney Injury and Drug-Induced Renal Injury

Acute kidney injury (AKI) is a fairly common disorder, albeit with high morbidity and mortality. In a rat ischemia-reperfusion model (bilateral renal artery clamping), alterations in the expression of 18 genes were identified by DNA microarray analysis. The *vnn1* gene was one of nine genes found to be upregulated in the early phase of AKI [64].

Hosohata et al. explored the potential of vanin 1, present at the brush borders where the first filtration occurs, as a biomarker for nephrotoxicant-induced renal injury [65]. They used ethylene glycol (EG) to cause nephrotoxicity in in vitro and in vivo models. EG is a well-known organic toxic compound that causes damage to the proximal tubuli due to crystallization and consequent obstruction [66,67]. Upon exposure to EG, vanin 1 mRNA expression and protein levels both increased in human renal tubular HK-2 cells and in an in vivo rat model. Moreover, in the EG-treated group, higher concentrations of soluble vanin 1 were observed both in serum and in urine [65].

Similarly, increased urinary vanin 1 levels were observed in rats intraperitoneally injected with cisplatin or gentamicin sulfate to induce nephrotoxicity [68]. While rats treated with EG showed higher vanin 1 mRNA levels [65], vanin 1 mRNA expression was not increased after cisplatin and gentamicin treatment. In addition, *vnn1* gene expression was evaluated in the human proximal tubular cell line HK-2. *vnn1* expression decreased in a dose-dependent manner after 24 h exposure to gentamicin or cisplatin. In contrast, upon exposure to EG, *vnn1* gene expression increased in the same HK-2 cell line. At the protein level, renal vanin 1 was also significantly decreased in rats treated with cisplatin and gentamicin. Thus, these different patterns at gene and protein levels of vanin 1 may be the result of the different mechanisms that lead to injury and toxicity. Cisplatin and gentamicin are known to directly cause renal cell death [69,70], whereas EG causes toxicity due to the accumulation of its metabolites. The Vanin 1 concentration in serum was found to be significantly higher in rats treated with EG compared to the control, while in rats treated with cisplatin and gentamicin its concentration was almost undetectable. Taken together, regardless of the toxicant causing injury, high concentrations of vanin 1 were found in the urine in both studies described above. Therefore, these results suggest a potential role for vanin 1 as a biomarker of renal injury.

#### 2.2.2. Vanin 1 in a Unilateral Ureteral Obstruction Model

Hydronephrosis can be one of the causes of chronic kidney disease. This swelling of the kidney is caused by a build-up of urine, which in turn is caused by urinary tract obstruction. Hosohata et al. [71] investigated the potential of vanin 1 as a biomarker in a unilateral ureteral obstruction (UUO) rat model, which is also used to develop renal fibrosis in mice and rats. On day 7 after UUO, urinary vanin 1 levels were significantly higher than those in urine from sham-operated rats, suggesting a potential cleavage of vanin 1 from the membrane or a higher expression of its soluble form. The levels of urinary vanin 1 in UUO remained high until day 14. There was no significant difference in the serum vanin 1 level between sham-operated rats and rats with UUO throughout the experiment. In kidney tissue of UUO rats, vanin 1 protein levels were significantly decreased only on day 14, while the vanin 1 mRNA level was significantly decreased already on day 3 and 7. Again, urinary vanin 1 was marked as a promising candidate biomarker for renal tubular injury, in this case due to hydronephrosis.

In our own work, we observed a similar downregulation of mRNA levels of vanin 1 in a UUO mouse model (Bartucci R, Salvati A, Olinga P, Boersma YL. Regulation of vanin 1 in precision-cut tissue slices ex vivo model as onset and end-stage disease models of fibrosis. Manuscript in preparation). The vanin 1 mRNA level was also decreased on day 3 and 7, whereas no difference in protein expression was observed in vanin 1 immuno-stained kidney sections of UUO mice compared to the sections of negative control. Additionally, precision-cut kidney slices (PCKS) obtained from UUO mice and healthy kidneys were maintained in culture for 48 h, after which downregulation of mRNA level of vanin 1 was observed.

Miyagawa and coworkers [72] analyzed human vanin 1 in samples collected from 28 hydronephrosis cases. In brief, they compared these 28 samples to those of 21 control patients and showed that vanin 1 concentration was, once again, really high in renal pelvic urine. Moreover, they demonstrated that vanin 1 and *N*-acetyl-β-d-glucosaminidase (NAG) were useful factors to predict hydronephrosis. Furthermore, they also studied the possibility to use vanin 1 as a biomarker to monitor the course after an upper urinary tract obstruction intervention and it was demonstrated that the levels of vanin 1 decreased after surgery. Thus, this pilot study demonstrates the better diagnostic value of renal pelvic vanin 1 in human samples than kidney injury molecule-1 (KIM-1) or neutrophil gelatinase-associated lipocalin (NGAL), commonly used as biomarkers for hydronephrosis conditions, and the potential of vanin 1 as biomarker for AKI was again confirmed.

#### 2.2.3. Vanin 1 in Diabetic Nephropathy

Vanin 1 was also indicated to play a role in kidney damage in a rat model of type 1 diabetic nephropathy (DN) [73]. Rats with streptozotocin-induced DN showed a most significant upregulation of vanin 1 at the protein level. This upregulation of vanin 1 in type 1 diabetes (T1D) was also seen in pooled urine of T1D patients with macroalbuminuria [74]. Nevertheless, the analysis of spot urine from individual patients showed great variability in vanin 1 concentration, without upregulation in DN patients.

In conclusion, these studies show that the renal expression of vanin 1, at both the gene and protein level, is modulated differently depending on the specific etiology of the injury and potential correlation with diseases such as diabetes. However, a common finding appears to be the significantly increased concentration of vanin 1 in urine collected in pathophysiological conditions, highlighting vanin 1′s potential as a valuable biomarker for renal diseases.

### 2.3. Intestine

Intestinal vanin 1 is mainly expressed by enterocytes at the brush border of the intestine, similar to the renal proximal tubuli [75]. While pantothenic acid is present in most foods as part of CoA, in this form it cannot be adsorbed by the enterocytes in the gut. However, pantothenic acid itself can easily pass the barrier of gut epithelial cells. Therefore, the presence of vanin 1 could be explained by its primary role to recycle Vitamin B5, which is necessary for the formation of CoA in the gut.

#### 2.3.1. Vanin 1 in Intestinal Inflammation Models

As described above, the strict correlation between vanin 1 and oxidative stress is well known. To assess a potential role for vanin 1 in intestinal pathologies and to examine the susceptibility of *vnn1* KO mice to intestinal inflammation, both acute and chronic infections were studied [75]. In animal models, oxidative stress in the gut can be generated by acute exposure to a nonsteroidal anti-inflammatory drug (NSAID), such as indomethacin, or a chronic infection with *Schistosoma mansoni*, a water-borne parasite. Although the *Schistosoma* model is largely used to better understand the delayed hypersensitivity response in liver [76], the intestine is known to be the second main site of egg deposition injury.

Histological examination of *vnn1* KO mice treated with indomethacin showed that the integrity and architecture of the intestinal tissue remained intact, in contrast to that of WT indomethacin-treated mice. Furthermore, *vnn1* KO mice showed less inflammation upon indomethacin treatment than WT mice. The iNOS and COX-2 mRNA levels were higher in WT than in *vnn1* KO mice.

Upon *Schistosoma* infection, *vnn1* KO mice survived longer than WT and detailed histological analyses showed that myeloid cell infiltration and tissue damage were more pronounced in WT than in *vnn1* KO mice. Interestingly, higher GSH levels were found in *vnn1* KO mice than in WT mice. These increased GSH levels in *vnn1* KO mice could be the result of enhanced synthesis in the liver, as indicated by an increase in γ-GCS activity. However, basal γ-GCS activity was comparable for *vnn1* KO and WT mice. In conclusion, in both models *vnn1* KO mice were shown to better control and survive intestinal inflammation and the ensuing duodenal hemorrhages that normally lead to death [75]. Collectively, these observations point to a pro-inflammatory role of vanin 1, with its pantetheinase activity as a major regulator of intestinal inflammation.

#### 2.3.2. Vanin 1 in a Colitis Mouse Model

Using a 2,4,6-trinitrobenzene sulfonic acid (TNBS)-colitis mouse model it was reported that vanin 1 deficiency protects mice from colitis [40]. This was clearly demonstrated by an improved survival of *vnn1* KO mice compared to WT animals after TNBS treatment. A total of 70% of *vnn1* KO mice survived the first ten days of treatment, whereas 80% of the WT animals died. This protection was reversible by administration of either cystamine or bisphenol A diglycidyl ether, a PPAR-γ antagonist. Vanin 1, by antagonizing PPAR-γ, licenses the production of inflammatory mediators by intestinal epithelial cells. Again, in chronic inflammation of the intestine vanin 1 acts as pro-inflammatory agent [40].

#### 2.3.3. Vanin 1 in Human Inflammatory Bowel Disease

Inflammatory bowel disease (IBD) is a chronic inflammatory syndrome of the digestive tract, which probably develops due to an immune system malfunction, while other factors, such as diet and stress, can aggravate it [77,78]. Since vanin 1 has been described as a sensor of stress, controlling oxidative stress and inflammation responses in many tissues, a role for vanin 1 in IBD was anticipated [39,79]. Not surprisingly, the *vnn1* transcript was found to be increased up to 400-fold, not only in IBD patients, but particularly in ulcerative colitis patients. Enhanced vanin 1 expression was detectable in biopsies harvested during the quiescent phase of the disease, suggesting that the overexpression is not necessarily correlated to inflammation. As single-nucleotide polymorphisms of *vnn1* have been shown to be important in certain pathologies before [80,81], a genetic association between *vnn1* and IBD was explored. Three single-nucleotide polymorphisms (SNPs) were found to be associated with IBD. These regions were correlated with *vnn1* transcript abundance in the colon. All in all, these findings suggest that vanin 1 could pose as a therapeutic target or fecal biomarker in IBD.

### 2.4. Lungs

Whether vanin 1 plays a (patho)physiological role in lung tissue remains an unanswered question, although vanin 1 expression in pulmonary tissue has been reported [82].

#### Vanin 1 in Asthma

In a recent study, vanin 1 was found to be a biomarker of corticosteroid treatment response in children with asthma [82]. Nasal epithelial cells, known to reflect the changes that appear in the bronchial airways in asthma patients, were sampled during asthma attacks. Genome-wide expression profiling demonstrated that vanin 1 mRNA expression consistently discriminated between good and poor responders to systemic corticosteroid treatment. Indeed, vanin 1 mRNA levels were significantly lower in the poor responder group compared to the good responder group. In addition, methylation levels at the chondroitin proteoglycan 4 (CpG4) site were significantly decreased in poor responders, while they were increased in good responders following treatment. These findings suggest that CpG4 methylation could regulate vanin 1 expression and, consequently, the response to corticosteroid treatment. To corroborate these results, *vnn1* KO mice were exposed to intratracheal doses of house dust mite (HDM) to induce allergic airway inflammation and treated with intraperitoneal dexamethasone. Airway hyperresponsiveness (AHR) was assessed after 24 h and bronchoalveolar lavage fluid (BALF) was collected and processed. Dexamethasone treatment significantly alleviated AHR in WT mice, as reflected by a 70% reduction in total BALF cells. In contrast, *vnn1* KO mice were unresponsive to dexamethasone treatment, demonstrating that vanin 1 contributes to an optimal response to corticosteroid treatment in experimental asthma [82]. Importantly, vanin 1 was not essential to asthma development, because *vnn1* KO mice did develop an asthma phenotype, including airway inflammation. However, further investigation is needed in order to better understand the specific role of vanin 1 in the lungs and correlated diseases, such as fibrosis.

## 3. Conclusions

The correlation of vanin 1, via its enzymatic production of cysteamine, with oxidative stress and consequently chronic inflammation is now well understood. Furthermore, vanin 1 partly regulates metabolic pathways through PPAR-α and PPAR-γ, thus playing a role in specific metabolic pathways. Vanin 1 is central in a plethora of pathological conditions, as described above in detail. Additionally, vanin 1 also plays a role in malaria susceptibility [6], psoriasis [83], carcinogenesis [84], cardiovascular disease [85], pediatric immune thrombocytopenia [86], and systemic sclerosis [21].

In certain diseases vanin 1 is upregulated, contributing to the progression and severity of the pathological status (Table 1). This raises the question of whether vanin 1 has potential as a biomarker of disease progression. Several studies have reported that vanin 1 is a direct PPAR-α target gene and that its serum protein level accurately reflects PPAR-α activation in the liver. In addition to the catabolism of FFAs, PPAR-α also modulates cytoprotection, inflammation, and hepatocarcinogenesis [87]. Thus, using serum vanin 1 as a biomarker may be of interest to monitor PPAR-α activity in liver disease.

In AKI, the potential of vanin 1 has been explored in more detail, particularly since vanin 1 is secreted in higher concentrations in urine of patients (Table 1). As such, vanin 1 is readily available, non-invasive, and easily measured, all characteristics of a good biomarker [88]. In addition, it was shown that vanin 1 could be measured with high sensitivity and specificity in human renal pelvic urine from patients with obstructive nephropathy. Furthermore, in patients receiving treatment, vanin 1 levels decreased significantly from one week after therapeutic intervention [72]. Hence, urinary vanin 1 is a promising biomarker, not only to detect, but also to monitor the clinical course of obstructive nephropathy (Table 1). Nevertheless, the precise role of vanin 1 in renal pathologies, be it protecting from or exacerbating inflammation, is unclear.

The development of *vnn1* KO mice has been pivotal for our understanding of vanin 1′s (patho)physiological role. Though the KO mice do not have an obvious spontaneous phenotype, they are resistant to inflammation and oxidative stress, thus indisputably proving a correlation between vanin 1, its pantetheinase activity and pro-inflammatory mediators (Table 1) [19,59]. Vanin 1 inhibitors have also been valuable tools in the elucidation of vanin biology. The first inhibitors showed IC_50_ values between 4–20 μM; however, their specificity was rather low [89]. Schalkwijk and coworkers developed a pantetheine analog, RR6, which showed high specificity towards vanin 1 [90]. The compound competitively and reversibly inhibited pantetheinase activity at nanomolar concentration; its potency was also confirmed in in vivo rat models [33,49,91]. Though further in-depth studies are warranted to specify the relevance of vanin 1 inhibition, compound RR6 certainly forms an exciting starting point to advance our knowledge of vanin biology and may lead to new therapeutic anti-inflammatory strategies.

## Figures and Tables

**Table 1:** Role of vanin 1 in diseases of organs in which it is highly expressed.

Organ.	Disease	Vanin 1
Liver	Steatosis	Higher transcription of gene expression [33,38,49]
	NAFLD/NASH	Promoting MP uptake by HSCs and endothelial cells [44,45]
	Hepatoxicity (APAP and CCl_4_)	Protective role [59,60,61]
Kidneys	AKI/drug-induced kidney injury/hydronephrosis and fibrosis/DN	Urinary biomarker [64,65,68,71,72]
Intestine	Intestinal inflammation/colitis/IBD	Pro-inflammatory role [40,75,76] Intestinal biomarker [39]
Lungs	Asthma	Biomarker as discriminating factor to corticosteroid treatment response [82]
